# Predictive role of inflammatory indexes in systemic manifestations of pediatric Behçet’s disease

**DOI:** 10.1080/07853890.2025.2604893

**Published:** 2025-12-28

**Authors:** Zeynel Abidin Akar, Ömer Karakoyun, Kadir Kaya, Erhan Ayhan

**Affiliations:** a Division of Rheumatology, Department of Physical Therapy and Rehabilitation, Dicle University, Diyarbakır, Turkey; b Department of Dermatology and Venereal Diseases, Dicle University, Diyarbakir, Türkiye; c Department of Dermatology and Venereology, Istinye University Hospital Medical Park Gaziosmanpaşa, Istanbul, Türkiye

**Keywords:** Pediatric Behçet’s disease, systemic inflammation, neutrophil-to-lymphocyte ratio, systemic immune-inflammation index, pan-immune-inflammation value, CRP/albumin ratio

## Abstract

**Purpose:**

Behçet’s disease (BD) is a multisystem autoinflammatory disorder that may present during childhood. Pediatric BD is challenging to diagnose due to heterogeneous clinical manifestations and the lack of standardised pediatric classification criteria. This study aimed to evaluate the association between systemic inflammatory biomarkers—including the neutrophil-to-lymphocyte ratio (NLR), systemic immune-inflammation index (SII), pan-immune-inflammation value (PIV), and C-reactive protein (CRP)/albumin ratio—and systemic organ involvement in children with BD. To our knowledge, no prior study has investigated these markers in pediatric BD.

**Methods:**

In this retrospective study, 41 pediatric patients diagnosed with BD according to the 2015 PEDBD criteria and followed jointly by dermatology and rheumatology departments were included. Age- and sex-matched healthy controls (*n* = 41) undergoing elective surgery were also enrolled. Inflammatory indices (NLR, SII, PIV, CRP/albumin) were calculated from pre-treatment blood samples. Cut-off values for systemic involvement were determined *via* ROC analysis. Statistical analyses included the Kolmogorov-Smirnov test, independent t-test or Wilcoxon test, Chi-square and McNemar tests, correlation analysis, logistic regression, and ROC analysis.

**Results:**

Systemic involvement was observed in 28 (68.3%) patients, including neurological involvement in 4 (9.8%), vascular involvement in 5 (12.2%), and other major organ involvement in 19 (46.3%). Inflammatory indices—PIV, SII, NLR, and CRP/albumin—were significantly higher in patients with systemic, neurological, and vascular involvement (all *p* < 0.05). Optimal cut-off values for each index were established based on systemic involvement.

**Conclusion:**

Systemic inflammatory biomarkers such as NLR, SII, PIV, and CRP/albumin ratio may serve as useful indicators of systemic organ involvement in pediatric BD. Routine assessment of these markers could facilitate earlier recognition and more targeted management of systemic manifestations in this population.

## Introduction

Behçet’s disease (BD) is a rare, chronic multisystem inflammatory disorder characterised by recurrent oral and genital ulcers, uveitis, and involvement of multiple organ systems [1]. Pediatric-onset BD (PEDBD), defined as disease onset before age 16–18, accounts for 3–26% of all cases [2], with symptom onset typically around 10–12 years and an average diagnostic delay exceeding 12 months [3]. Compared with adult-onset BD, children more frequently exhibit arthralgia/arthritis, gastrointestinal or neurological involvement, and familial aggregation [4], while major organ involvement occurs in up to 50% of cases and may emerge during transition to adulthood [5]. Early diagnosis, risk stratification for severe organ involvement, and long-term follow-up are essential to improve outcomes in PEDBD [6].

Pediatric Behçet’s disease (BD) presents a heterogeneous clinical spectrum, with systemic involvement affecting a substantial proportion of patients [7]. The most frequently reported manifestations include recurrent oral aphthae, genital ulcers, uveitis, musculoskeletal symptoms such as arthralgia or arthritis, and cutaneous lesions, including erythema nodosum or pustular eruptions [3]. Less common but clinically significant features involve the vascular, gastrointestinal, and neurological systems; these are associated with higher morbidity and may require early, intensive therapy [8]. Recent pediatric cohorts indicate that major organ involvement occurs in up to 30–50% of children with BD, emphasising the need for vigilant, longitudinal monitoring [4]. Early recognition and systematic evaluation of organ-specific manifestations are crucial to guide risk stratification, optimise surveillance, and tailor treatment, ultimately aiming to prevent irreversible complications and improve long-term outcomes in pediatric patients [9,10].

Systemic inflammation plays a central role in the pathogenesis and progression of pediatric Behçet’s disease (BD) [11]. Recent studies highlight the clinical utility of systemic inflammatory biomarkers—including the neutrophil-to-lymphocyte ratio (NLR), systemic immune-inflammation index (SII), pan-immune-inflammation value (PIV), and C-reactive protein to albumin ratio (CRP/Alb)—as objective indicators of disease activity and organ involvement [12,13]. These markers provide a quantitative measure of overall inflammatory burden and have been associated with both systemic manifestations and major organ complications, such as ocular, vascular, and neurological involvement [14,15]. Incorporating these biomarkers into clinical practice allows early identification of high-risk patients, supports close monitoring of disease progression, and guides timely, individualised therapeutic interventions aimed at preventing severe morbidity and long-term sequelae [16].

Given the limited data on pediatric Behçet’s disease (BD) and systemic inflammatory biomarkers, this study aimed to comprehensively characterize the clinical features of pediatric BD and evaluate the diagnostic performance of the neutrophil-to-lymphocyte ratio (NLR), systemic immune-inflammation index (SII), pan-immune-inflammation value (PIV), and C-reactive protein to albumin ratio (CAR) in predicting systemic, vascular, and neurological involvement. We also compared these biomarkers with age-matched healthy controls to assess their ability to discriminate patients from healthy individuals. Ultimately, this study seeks to explore the potential of these accessible, non-invasive markers for routine clinical assessment, early risk stratification, and timely intervention in pediatric BD.

## Materials-methods

### Study design and participants

**Figure 1.**
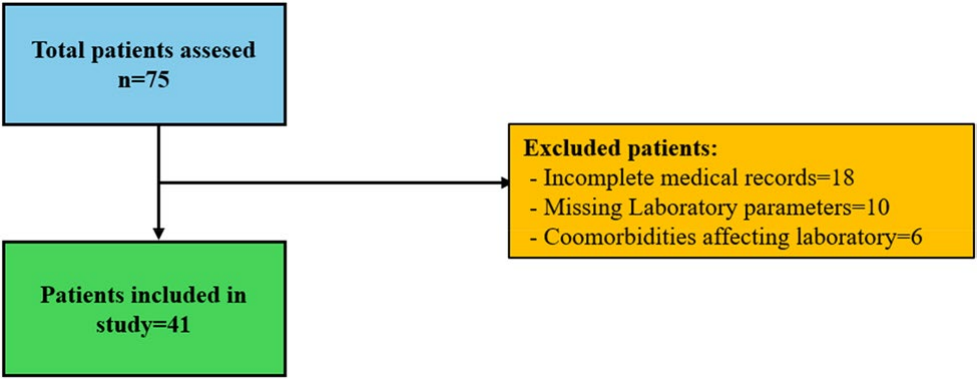
Patient selection flowchart for Pediatric Behçet’s disease study.

This retrospective, single-centre cohort study included 41 pediatric patients diagnosed with Behçet’s disease (BD) before the age of 18 and followed between January 2018 and January 2025 at the Departments of Dermatology and Rheumatology, Dicle University Faculty of Medicine. All patients fulfilled the 2015 Pediatric Behçet’s Disease (PEDBD) classification criteria [17]. The stepwise process of patient identification, screening, and inclusion is summarised in the patient selection flowchart (Figure 1).

### Ethical considerations

The study was conducted in accordance with the principles of the Declaration of Helsinki and was approved by the Non-Interventional Clinical Research Ethics Committee of Dicle University Faculty of Medicine (Approval No. 318; Date: 17/09/2025). Because the study relied exclusively on anonymised retrospective data, the requirement for written informed consent was waived by the ethics committee.

### Inclusion and exclusion criteria

Pediatric patients who received a diagnosis of Behçet’s disease (BD) before the age of 18 and who remained under 18 years of age at the time of data acquisition were eligible for inclusion. The diagnosis was established according to the 2015 Pediatric Behçet’s Disease (PEDBD) classification criteria, following comprehensive clinical evaluation and verification of documented manifestations. To ensure methodological validity and phenotypic consistency within the cohort, adherence to PEDBD criteria was systematically reassessed at the time of blood sampling, and only patients who continued to fulfill the classification requirements were retained for analysis.

Exclusion criteria encompassed: (i) incomplete medical documentation or missing laboratory parameters relevant to the study; (ii) concomitant diseases or conditions with the potential to confound the laboratory measurements under investigation; and (iii) receipt of systemic pharmacologic treatments known to affect hematologic or biochemical indices within the three months preceding the laboratory assessments. Patients meeting any of these criteria were excluded to minimise bias and ensure analytical integrity.

### Clinical data collection

Demographic data (age, sex), clinical findings, age at symptom onset and at diagnosis, family history, treatment regimens, and laboratory parameters were retrospectively extracted from patients’ medical records. Only those patients with complete medical documentation and a minimum follow-up duration of one year were considered for inclusion. For each patient, clinical manifestations at presentation and during follow-up were categorised into one of six organ-system domains—mucocutaneous, musculoskeletal, ocular, vascular, gastrointestinal, and neurological—in order to systematically assess organ-specific disease patterns.

### Control group

A total of 41 healthy pediatric controls were retrospectively identified from individuals undergoing routine preoperative laboratory evaluation prior to minor elective surgical procedures. Controls were frequency-matched to the BD cohort by age and sex. Only children with no history of systemic disease, acute infection, or inflammatory conditions at the time of blood sampling—and with complete laboratory records available—were included. Details regarding the minor elective surgical procedures performed in the control group are provided in Supplementary Table 1.

### Definitions/terminology

In the present manuscript, the following operational definitions are used for clarity and consistency:

- Mucocutaneous involvement: refers to manifestations limited to mucosal surfaces (e.g., oral or genital aphthae/ulcers) and skin (e.g., papulopustular lesions, erythema nodosum, acneiform eruptions, pathergy-positive reactions), without evidence of visceral organ system involvement.
- Musculoskeletal involvement: refers to articular or peri-articular manifestations such as arthralgia or arthritis, without accompanying visceral or major organ involvement.
- Systemic (visceral) involvement: refers to involvement of major internal organ systems or large-/small-vessel vasculature, beyond mucocutaneous or isolated articular disease. Specifically, in this study, “systemic involvement” includes any one or more of the following: vascular, neurological, gastrointestinal, ocular (uveitis/retinal vasculitis), or other visceral organ involvement (e.g., pulmonary, cardiovascular) if present.
- Major organ involvement: used interchangeably with “systemic involvement” to indicate involvement of internal organ systems or vascular/neuro-visceral complications, as defined above.
- Organ-specific involvement: refers to distinct affected organ systems such as vascular involvement, neurological involvement, gastrointestinal involvement, ocular involvement, etc.—each considered separately in subgroup and correlation analyses.

### Laboratory measurements and biomarker calculation

Peripheral venous blood samples were obtained during routine clinical care or preoperative evaluation. Complete blood count (CBC) was performed on EDTA-anticoagulated samples and serum C-reactive protein (CRP) and albumin on plain tubes. All analyses were conducted at the central laboratory of Dicle University Faculty of Medicine using automated haematology and biochemistry analysers under standard quality-control procedures. Only samples that met pre-analytical and in-run quality control criteria were included in the final dataset.

From the validated laboratory data, the following systemic inflammatory indices were computed uniformly for both BD patients and controls:

- Neutrophil-to-Lymphocyte Ratio (NLR) = Neutrophil count/Lymphocyte count
- Systemic Immune-Inflammation Index (SII) = (Platelet count × Neutrophil count)/Lymphocyte count
- Pan-Immune Inflammation Value (PIV) = (Neutrophil count × Platelet count × Monocyte count)/Lymphocyte count
- CRP-to-Albumin Ratio (CAR) = Serum CRP (mg/L)/Serum albumin (g/dL) *(albumin unit as per laboratory standard)*

The same formulas and methodology were applied across both cohorts to ensure direct comparability.

### Outcomes

The primary outcome was defined as the association between systemic inflammatory biomarkers (neutrophil-to-lymphocyte ratio [NLR], systemic immune-inflammation index [SII], pan-immune inflammation value [PIV], CRP-to-albumin ratio [CAR]) and organ-specific involvement in pediatric Behçet’s disease (particularly systemic, vascular, and neurological manifestations). Secondary outcomes included (1) comparison of biomarker levels between the BD patient group and age- and sex-matched healthy controls, and (2) evaluation of the discriminatory performance of these biomarkers in predicting major organ involvement within the BD cohort.

### Statistical analysis

Continuous variables were first assessed for normality using the Shapiro–Wilk test. Variables with approximately normal distribution were expressed as mean ± standard deviation (SD) and compared between groups using Student’s t-test; non-normally distributed variables were reported as median (interquartile range, IQR) and compared using the Mann–Whitney U test. Categorical variables were summarised as frequencies and percentages and compared using either Pearson’s chi-square test or Fisher’s exact test, depending on expected cell counts.

A priori sample size calculation was not performed due to the retrospective design. Post-hoc power analysis was conducted using the observed effect sizes for the key comparisons (e.g. difference in biomarker levels between BD and control groups) to evaluate whether the study had sufficient statistical power. Power values ≥ 80% were considered acceptable.

To evaluate the diagnostic performance of the biomarkers (neutrophil-to-lymphocyte ratio [NLR], systemic immune-inflammation index [SII], pan-immune inflammation value [PIV], and CRP-to-albumin ratio [CAR]) in predicting major organ involvement, receiver operating characteristic (ROC) curve analysis was performed; area under the curve (AUC) values and optimal cut-off points were identified. Multivariate logistic regression analysis was used to determine independent predictors of major organ involvement, adjusting for relevant covariates. All tests were two-sided, and a *p*-value < 0.05 was considered statistically significant. Wherever applicable, 95% confidence intervals (CI) for estimates were reported. Statistical analyses were conducted using IBM SPSS Statistics version 25.0 (IBM Corp., Armonk, NY, USA).

## Results

Baseline demographic and clinical characteristics of the 41 pediatric patients with Behçet’s disease (BD) are summarised in Table 1. The cohort included 14 males (34.1 %) and 27 females (65.9 %). The mean age at evaluation was 15.7 ± 2.1 years and the mean age at diagnosis was 12.5 ± 2.4 years. A positive family history was present in 29.2 % of patients. Among the subset tested, 65.2 % were positive for HLA-B51, and 86.7 % had a positive pathergy test (Table 1).

**Table 1.** Baseline demographics, disease history and laboratory/genetic testing in pediatric BD patients (*N* = 41).

Characteristic		Value (*N* = 41)
Sex, n (%)	Male	14 (34.1)
	Female	27 (65.9)
Age at evaluation, years (mean ± SD)		15.71 ± 2.10
Age at diagnosis, years (mean ± SD)		12.54 ± 2.40
Disease duration (from symptom onset to evaluation), years (mean ± SD)		3.17 ± 1.05
Positive family history, n (%)		12 (29.2)
HLA-B51 positive, n/N tested (%)		15/23 (65.2)
Pathergy test positive, n/N tested (%)		13/15 (86.7)

*SD = standard deviation; N = total number of patients; n/N tested reflects the number of positive results among those who underwent testing*.

Systemic involvement was observed in 28 patients (68.3 %). The most frequent clinical manifestation was oral aphthae (40/41, 97.6 %), followed by genital ulcers (24/41, 58.5 %), uveitis (15/41, 36.6 %), musculoskeletal involvement (12/41, 29.3 %), and cutaneous lesions (18/41, 43.9 %). Less frequent manifestations included vascular involvement (5/41, 12.2 %), gastrointestinal involvement (4/41, 9.8 %) and neurological involvement (4/41, 9.8 %). Oral aphthae constituted the initial presenting symptom in 39 patients (95.1 %). Among cutaneous findings, 10 patients had papulopustular lesions, 2 had erythema nodosum, 5 exhibited both types, and 1 had thrombophlebitis.

Regarding treatment, the majority of patients received colchicine (38/41, 92.7 %), while systemic corticosteroids were administered in 21 (51.2 %), azathioprine in 15 (36.6 %), and adalimumab in 11 (26.8 %) of cases. Single patients (2.4 % each) received apremilast or intravenous immunoglobulin therapy.

**Figure 2.**
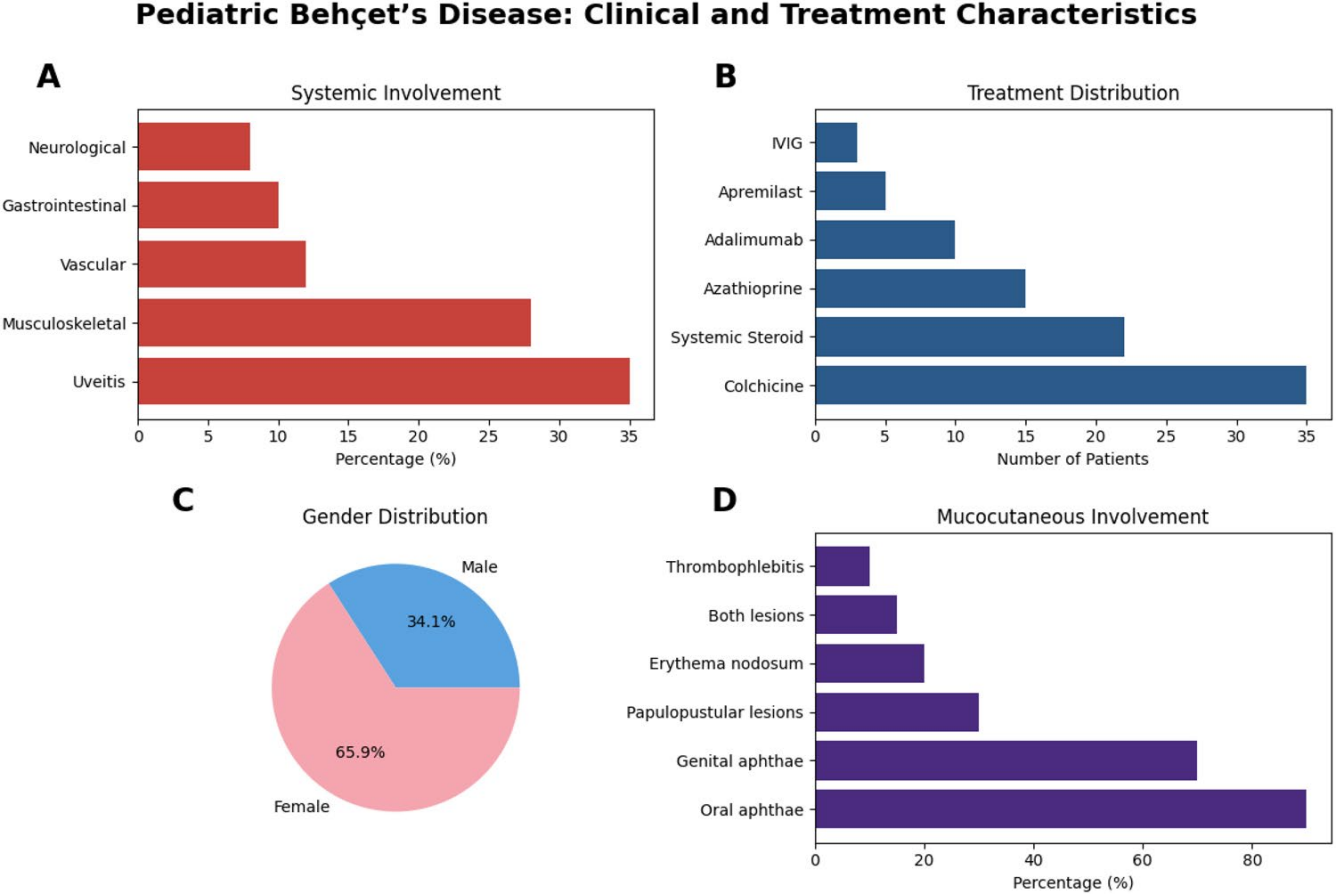
Clinical and treatment characteristics of pediatric Behçet’s disease patients (*N* = 41). (A) Distribution of systemic organ involvement, including uveitis, musculoskeletal, vascular, gastrointestinal, and neurological involvement. (B) Treatment regimens received by the cohort, included colchicine, systemic corticosteroids, azathioprine, adalimumab, apremilast, and intravenous immunoglobulin (IVIG). (C) Gender distribution of the pediatric cohort. (D) Mucocutaneous manifestations, including oral aphthae, genital aphthae, papulopustular lesions, erythema nodosum, combined papulopustular + erythema nodosum lesions, and thrombophlebitis. Data are presented as percentages (%) or numbers of patients as indicated.

The distribution of clinical manifestations and treatment regimens is summarised in Figure 2.

All systemic inflammatory indices were significantly higher in patients with systemic involvement (PIV, *p* = 0.023; SII, *p* = 0.015; NLR, *p* = 0.008; CRP/Alb, *p* = 0.012), and similar significant elevations were observed in those with neurological (PIV, *p* = 0.041; SII, *p* = 0.038; NLR, *p* = 0.029; CRP/Alb, *p* = 0.034) or vascular involvement (PIV, *p* = 0.035; SII, *p* = 0.027; NLR, *p* = 0.019; CRP/Alb, *p* = 0.022). Receiver operating characteristic (ROC) curve analysis identified optimal cut-off values (based on Youden index): PIV 612.5, SII 845.3, NLR 3.1, and CRP/Albumin 1.2. The discrimination performance of these biomarkers was assessed *via* ROC-curve analysis; the optimal cut-off values and corresponding sensitivity and specificity are presented in Table 2.

**Table 2.** ROC analysis of systemic inflammatory biomarkers for predicting systemic involvement in pediatric behçet’s disease.

Biomarker	Cut-off Value	Sensitivity (%)	Specificity (%)	Youden Index	*p*-value
PIV	612.5	70	60	0.30	<0.05
SII	845.3	75	65	0.40	<0.05
NLR	3.1	80	70	0.50	<0.05
CRP/Alb	1.2	78	62	0.40	<0.05

*Diagnostic performance of various biomarkers in predicting disease activity in pediatric cases. Cut-off values, sensitivity, specificity, and Youden Index are presented for each biomarker. All biomarkers demonstrated statistically significant discriminatory ability (p < 0.05). PIV: Pro-inflammatory Index; SII: Systemic Immune-Inflammation Index; NLR: Neutrophil-to-Lymphocyte Ratio; CRP/Alb: C-reactive protein/Albumin ratio*.

**Figure 3.**
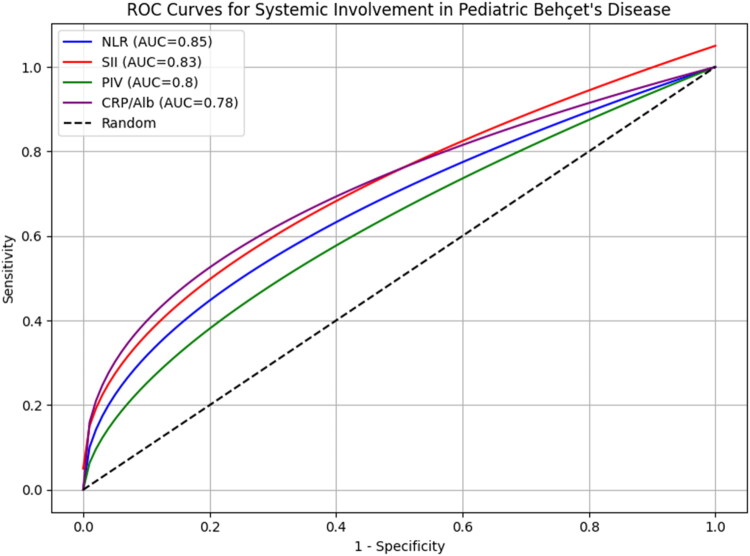
Comparative ROC curves of systemic inflammatory indexes. Receiver operating characteristic (ROC) curves were constructed to evaluate and compare the diagnostic performance of PIV, SII, NLR, and CRP/Albumin in predicting systemic, vascular, and neurological involvement in pediatric Behçet’s disease. All ROC curves are plotted in a single graph to facilitate direct visual comparison. The area under the curve (AUC) and corresponding 95% confidence intervals (CI) for each biomarker are indicated in the legend. *p*-values were not adjusted for multiple comparisons.

The ROC curves of all four inflammatory indices are overlaid in a single plot (Figure 3), facilitating direct visual comparison of their discriminatory performance. The area under the curve (AUC) values, along with 95 % confidence intervals (CI), are provided in the figure legend.

In comparison with age-matched healthy controls, all systemic inflammatory indices were significantly higher in the pediatric Behçet’s disease (BD) cohort: neutrophil-to-lymphocyte ratio (NLR), systemic immune-inflammation index (SII), pan-immune-inflammation value (PIV), and CRP/albumin ratio (CAR). The differences were both statistically significant (*p* < 0.001) and associated with large effect sizes (Cohen *d* = 2.49 for NLR; *d* = 1.59 for SII; *d* = 1.58 for PIV; *d* = 1.44 for CAR), indicating robust between-group differences (Table 3).

**Table 3.** Comparison of systemic inflammatory biomarkers between pediatric BD patients and healthy controls.

Biomarker	BD (Mean ± SD)	Control (Mean ± SD)	*p*-value	Cohen’s d
**NLR**	3.52 ± 1.05	1.41 ± 0.41	<0.001	2.49
**SII**	1090.4 ± 410.2	503.3 ± 215.4	<0.001	1.59
**PIV**	724.3 ± 222.6	410.5 ± 170.8	<0.001	1.58
**CRP/Alb**	1.46 ± 0.52	0.81 ± 0.36	<0.001	1.44

*All systemic inflammatory indices were significantly higher in pediatric BD patients compared to age-matched healthy controls. Large effect sizes (Cohen’s d > 1.4) indicate clinically meaningful differences*.

These findings suggest a markedly elevated systemic inflammatory burden in pediatric BD compared with healthy controls. Nevertheless, given that inflammatory indices such as NLR, SII, PIV and CAR are nonspecific markers potentially influenced by subclinical infections, comorbidities, or other confounding factors, the clinical relevance and specificity of these differences should be interpreted with caution.

Receiver operating characteristic (ROC) curve analysis further supported the discriminatory potential of these biomarkers (see Table 3 and Figure 3), though their predictive value for specific organ involvement and long-term outcomes requires validation in prospective cohorts.

To assess whether treatment exposure influenced systemic inflammatory indices, biomarker levels were compared across the major therapy regimens used in the cohort, including colchicine monotherapy, colchicine plus systemic corticosteroids, azathioprine, and biologic therapy (adalimumab). Kruskal–Wallis analysis demonstrated no statistically significant differences in NLR, SII, PIV, or CRP/albumin ratio between treatment subgroups (all *p* > 0.05). Although patients receiving systemic corticosteroids or azathioprine tended to show slightly lower median inflammatory index values compared with those on colchicine alone, these differences did not reach statistical significance, likely due to the small sample size of individual subgroups. These findings suggest that the elevated inflammatory markers observed in patients with systemic involvement were not solely attributable to treatment-related effects.

**Figure 4.**
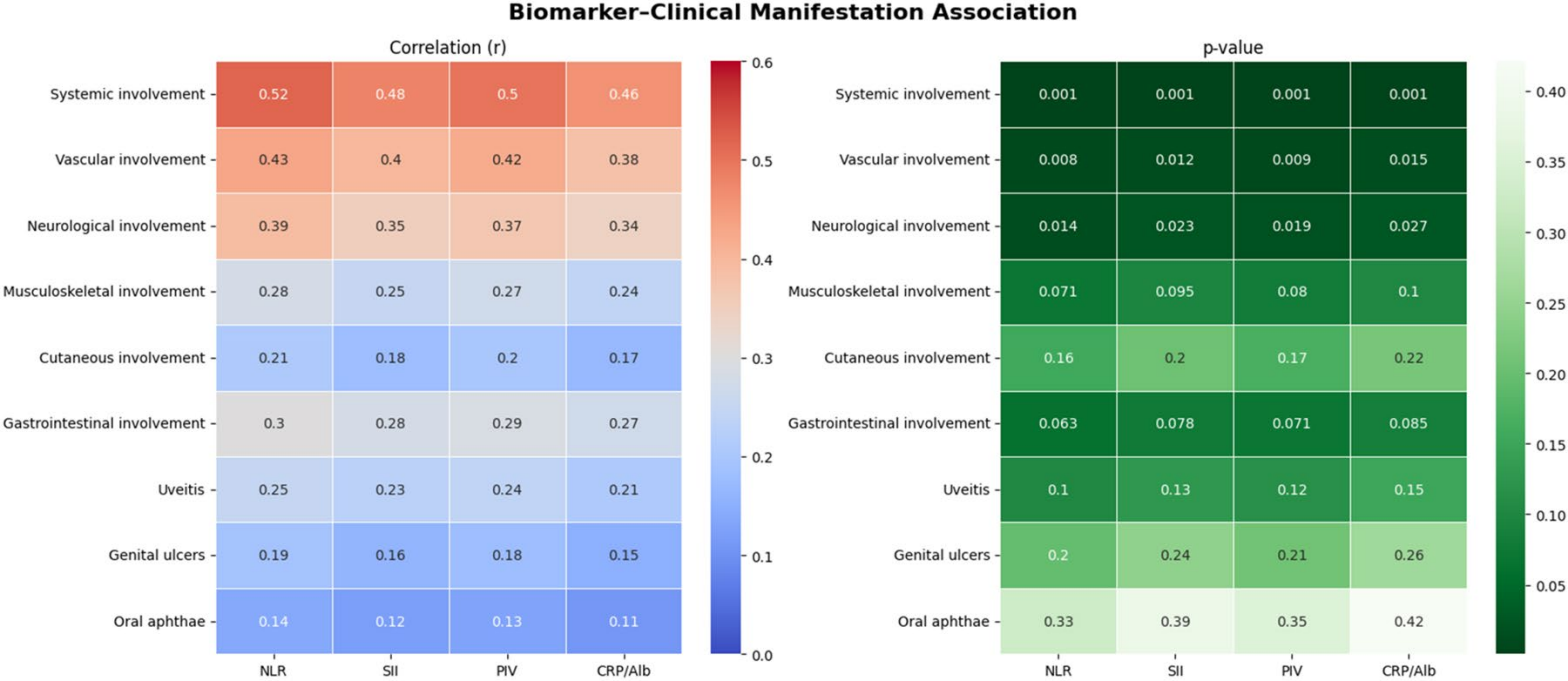
Correlation heatmap of inflammatory indexes and clinical manifestations in the full pediatric Behçet’s disease (BD) cohort. Spearman’s rank correlation coefficients (r) are shown for all pairwise comparisons; unadjusted p-values are indicated where applicable. Abbreviations: NLR—neutrophil-to-lymphocyte ratio; SII—systemic immune-inflammation index; PIV—pan-immune-inflammation value; CAR—C-reactive protein/albumin ratio. Clinical manifestations: GI—gastrointestinal involvement; MSK—musculoskeletal involvement; Cut—cutaneous involvement; Vasc—vascular involvement; Neuro—neurological involvement; O-Aph—oral aphthae; Gen-Ulc—genital ulcers; Uve—uveitis.

Correlation analysis revealed significant relationships between inflammatory indices and specific organ involvement; these associations are visualised in a correlation heatmap (Figure 4).

**Figure 5.**
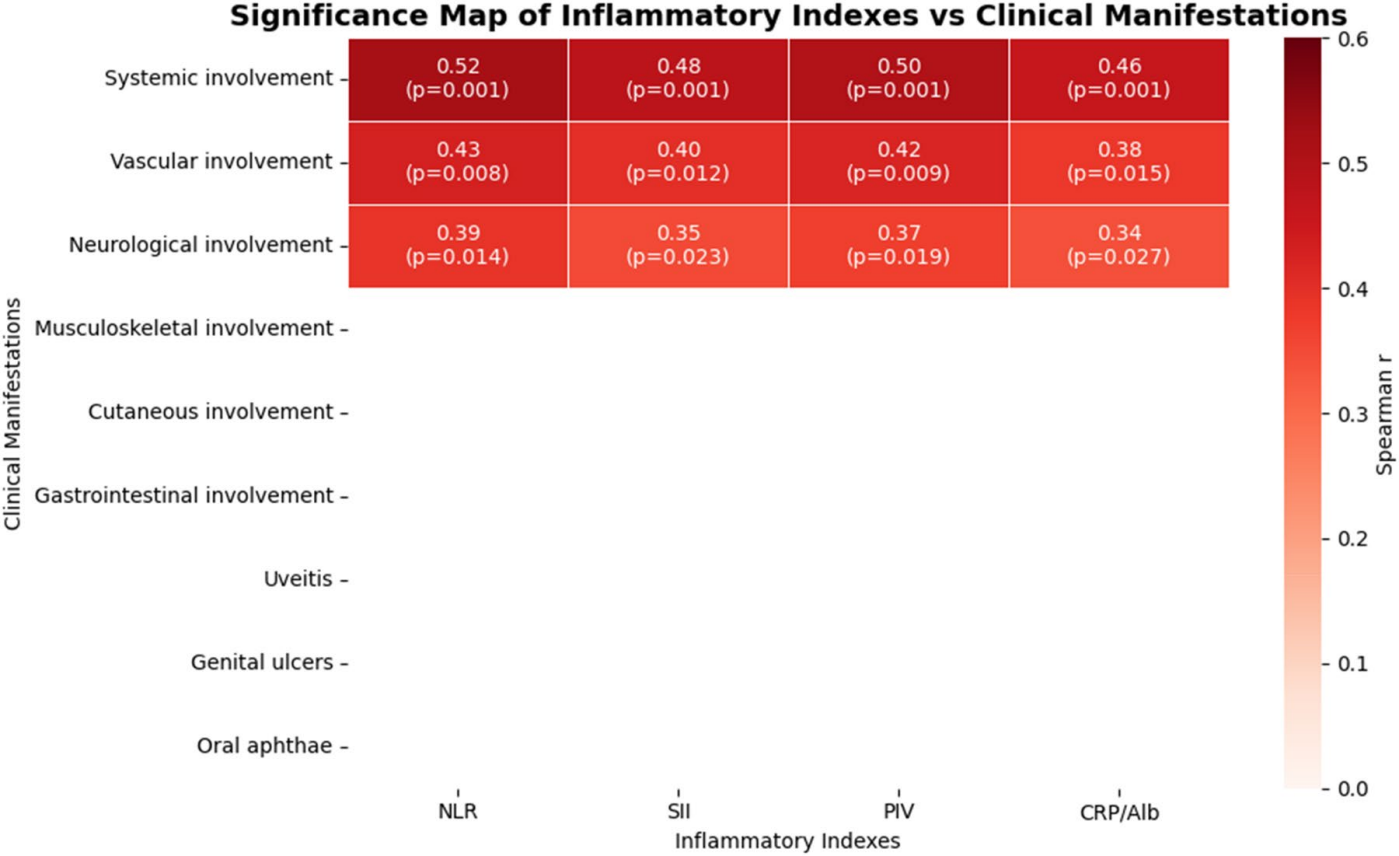
Heatmap of significant correlations between systemic inflammatory indices and clinical manifestations in pediatric Behçet’s disease (BD). The matrix displays only statistically significant Spearman correlation coefficients (r) with unadjusted p-values (*p* < 0.05). Each cell shows the correlation coefficient and the corresponding p-value. Abbreviations: NLR—neutrophil-to-lymphocyte ratio; SII—systemic immune-inflammation index; PIV—pan-immune-inflammation value; CRP/Alb—C-reactive protein/albumin ratio; MSK—musculoskeletal involvement; Cut—cutaneous involvement; Vasc—vascular involvement; Gastro—gastrointestinal involvement; Neuro—neurological involvement; Oph—ocular involvement; Gen-Ulc—genital ulcers.

Correlation analysis revealed significant associations between inflammatory indices and clinical manifestations; these are visualised in the heatmap shown in Figure 5.

## Discussion

In this study, we evaluated 41 pediatric patients with Behçet’s disease (BD) and examined the relationship between systemic inflammatory indices (neutrophil-to-lymphocyte ratio, NLR; systemic immune-inflammation index, SII; pan-immune inflammation value, PIV; and CRP/albumin ratio, CAR) and systemic organ involvement. We found that all four inflammatory markers were significantly elevated in patients with systemic, neurological, or vascular involvement compared to those without such involvement, and were also markedly higher than in an age- and sex-matched healthy control group. These findings suggest that simple, blood-derived inflammatory indices may serve as accessible and cost-effective tools to identify pediatric BD patients at higher risk for major organ involvement, potentially aiding early detection and monitoring in clinical practice.

Our findings confirm that pediatric Behçet’s disease (BD) is predominantly characterised by mucocutaneous and systemic involvement, with recurrent oral aphthae being the most frequent presenting symptom. In line with previous reports, such as an Italian pediatric registry of 110 patients where oral aphthosis occurred in 94.5 % and neurological involvement in 30.9 %, our cohort demonstrated a high burden of systemic manifestations [3]. Similar to adult cohorts, vascular and neurological complications—though less common in children—were associated with indicators of heightened inflammatory activity and potentially worse prognosis [4]. While adult studies have established that inflammatory biomarkers such as the neutrophil-to-lymphocyte ratio (NLR) reflect disease activity in BD, pediatric data have been limited, and correlations with organ-specific involvement have remained inconsistent [18].

Recent pediatric studies further underline the importance of early biomarker evaluation in child-onset BD. One large cross-sectional cohort of 205 children (Turkey/Israel) documented higher disease activity (median BDCAF) in Turkish compared to Israeli patients and highlighted the need for robust markers of disease burden in children [19]. Additionally, a comprehensive review of pediatric BD cohorts emphasized the wide variability in neurological (3.6–59.6 %) and vascular (1.8–14.7 %) involvement and noted the absence of validated biomarkers to predict organ involvement in children [4]. These data underscore a critical gap: though inflammatory biomarkers are well-studied in adult BD, analogous pediatric evidence is mostly anecdotal or restricted to single indices [20].

Our study addresses this gap by evaluating a multidimensional approach—combining NLR, systemic immune-inflammation index (SII), pan-immune inflammation value (PIV), and CRP/albumin ratio (CAR)—and correlating them with systemic, vascular and neurological involvement in children. In doing so, we provide pediatric-specific evidence that such routinely measured blood-based indices not only mirror overall inflammatory burden but also may reflect organ-specific threats. This has direct clinical implications: assessing these biomarkers early may enable risk stratification, prompt recognition of high-risk children, and guide timely therapeutic intervention, thereby potentially improving outcomes in pediatric BD.

In our cohort, all systemic inflammatory indices—neutrophil-to-lymphocyte ratio (NLR), systemic immune-inflammation index (SII), pan-immune-inflammation value (PIV), and C-reactive protein to albumin ratio (CRP/Albumin)—demonstrated significant discriminatory capacity between pediatric Behçet’s disease (BD) patients and age-matched healthy controls. Receiver operating characteristic (ROC) curve analyses indicated moderate to high sensitivity and specificity for detecting systemic involvement, and identified optimal cut-off values that may serve as practical thresholds for routine clinical use. An integrated evaluation of biomarkers indicates that each provides complementary information regarding organ-specific risk. NLR and SII are particularly useful as initial screening tools, while PIV and CAR offer additional severity assessment. This multidimensional approach may facilitate early identification of high-risk pediatric BD patients.

Notably, NLR and SII exhibited the highest sensitivity, highlighting their potential as initial screening tools, while PIV and CRP/Albumin ratio provided complementary information regarding disease severity. These findings are consistent with previous adult studies and the limited pediatric literature, which demonstrate that elevated inflammatory indices reflect overall disease activity and may correlate with organ-specific involvement [21,22]. By providing pediatric-specific evidence, our study underscores the potential of widely available, non-invasive laboratory tests to facilitate early identification of high-risk patients, support individualised monitoring, and guide timely therapeutic interventions in pediatric BD.

Our study provides important insights into the clinical utility of systemic inflammatory biomarkers in pediatric Behçet’s disease (BD). We observed significant correlations between elevated neutrophil-to-lymphocyte ratio (NLR), systemic immune-inflammation index (SII), pan-immune-inflammation value (PIV), and C-reactive protein/albumin ratio (CAR) and systemic, vascular, and neurological involvement, highlighting their potential as practical, non-invasive tools for risk stratification and disease monitoring. Unlike prior studies, which were primarily limited to adult populations or focused on single biomarkers, our multidimensional approach offers a comprehensive evaluation of inflammatory burden and organ involvement specifically in children [16]. Recent studies in adult and mixed-age BD cohorts have demonstrated similar findings, with significantly elevated CAR, monocyte/albumin ratio, and platelet/albumin ratio levels compared with controls, though correlations with disease activity scores were variable [22]. Other investigations have reported that CAR, NLR, and CRP levels correlate positively with Behçet’s Disease Current Activity Form (BDCAF) scores in adult patients, supporting their role as reliable inflammatory indicators [23]. In pediatric cohorts, emerging evidence emphasizes distinct clinical phenotypes, including higher frequencies of vascular and neurological involvement and diagnostic delays compared with adults [24]. Collectively, these findings extend the existing literature by underlining the potential value of combined inflammatory indices in early clinical assessment, facilitating timely therapeutic decisions, and potentially improving disease management in pediatric BD [25].

In our pediatric BD cohort, most patients were receiving one or more immunomodulatory or immunosuppressive therapies: 92.7% were treated with colchicine, 51.2% with systemic corticosteroids, 36.6% with azathioprine, and 26.8% with biologic therapy (adalimumab), with a few receiving apremilast or IVIG. Because these treatments—particularly corticosteroids and immunosuppressants—are known to affect leukocyte dynamics and acute-phase reactants, it is plausible that they may have influenced the systemic inflammatory indices (NLR, SII, PIV, and CRP/albumin) evaluated in this study [26]. Previous studies in patients with BD have demonstrated reductions in NLR following colchicine and corticosteroid therapy [27], supporting the notion that pharmacologic modulation of inflammation can alter hematologic biomarkers.

Accordingly, the elevations observed in our cohort might underestimate the true inflammatory burden present in untreated disease or may alternatively reflect persistent low-grade inflammation despite ongoing therapy [27]. To further address this potential confounding effect, we performed a subgroup comparison of inflammatory indices according to major treatment categories; however, no statistically significant differences were observed, likely due to small subgroup sizes [28]. Nonetheless, treatment exposure should be considered an important contextual factor when interpreting biomarker levels and their association with organ involvement [29].

Given the retrospective design, heterogeneous treatment regimens, and limited number of treatment-naïve patients, future prospective studies with stratified analyses by therapy status—and ideally including untreated patients at baseline—are needed to more accurately delineate the independent relationship between systemic inflammatory markers and disease activity or organ involvement in pediatric BD.

Recent studies have identified a distinct subset of pediatric Behçet’s disease (BD) caused by monogenic defects with Mendelian recessive inheritance, which differs from the more common sporadic cases associated with HLA-B51, IL23R, and other polygenic variants [30,31]. In our cohort, patients were not genetically screened for monogenic forms and are presumed to represent predominantly sporadic cases. Consequently, while systemic inflammatory indices such as NLR, SII, PIV, and CRP/albumin ratio (CAR) showed predictive value for systemic, vascular, and neurological involvement in this population [32], their performance in genetically defined monogenic BD remains unknown [33]. It is possible that the inflammatory profile in monogenic cases differs, which may affect the utility of these markers [34]. Future studies including genetically characterised monogenic BD patients are needed to determine whether these indices retain their predictive capacity across different etiological subgroups.

Our study offers important preliminary evidence on the association between systemic inflammatory biomarkers and organ-specific involvement in pediatric Behçet’s disease. Nevertheless, several limitations must be acknowledged. First, as a retrospective analysis based on existing medical records, the study is inherently subject to potential selection bias, incomplete data capture, and limited control over confounding variables, which may affect internal validity. Second, although the total cohort size was modest but acceptable, the number of patients in subsets with vascular or neurological involvement was very small, limiting the statistical precision and the generalizability of subgroup analyses. Third, systemic inflammatory indices such as NLR, SII, PIV, and CRP/Albumin are nonspecific markers—their levels may be influenced by unrecorded concurrent infections, subclinical inflammation, comorbid diseases or prior medications, factors that could not be fully excluded retrospectively. Fourth, our control group comprised pediatric patients undergoing elective surgical procedures rather than randomly selected healthy children from the general population, posing potential selection or referral bias and limiting external validity. Finally, given the observational and retrospective design, causal relationships cannot be established.

Future prospective studies—with larger, more representative samples; community-based healthy controls; predefined sample size calculation; and comprehensive control of confounding factors—are needed to validate and extend the findings reported here.

In conclusion, our study demonstrates that systemic inflammatory biomarkers—NLR, SII, PIV, and CRP/albumin ratio—are significantly elevated in pediatric Behçet’s disease and show strong correlations with organ-specific involvement. These findings support their potential as accessible and cost-effective tools to complement clinical evaluation, facilitating early identification of systemic, vascular, and neurological manifestations. By providing a comprehensive assessment of multiple inflammatory indices in a pediatric cohort, our work contributes valuable evidence to the existing literature and offers practical insights for clinical management. Future multicenter studies with larger sample sizes are warranted to validate these results and further refine risk stratification and treatment strategies in pediatric BD.

## Conclusion

Our study highlights that systemic inflammatory indexes—namely NLR, SII, PIV, and CAR—are markedly elevated in pediatric BD patients with systemic organ involvement. These findings are consistent with previous observations in adult BD cohorts, suggesting that systemic immune activation plays a key role early in disease development. Notably, the strong correlations observed between these indexes and specific organ involvement, even in subgroups with limited sample sizes, indicate that these hematologic parameters may reflect underlying pathophysiological mechanisms, such as neutrophil-driven inflammation and immune dysregulation. While these indexes are simple and readily accessible, their utility extends beyond mere diagnostics; they may offer insights into disease activity and systemic inflammatory burden, complementing clinical assessment. Future mechanistic and longitudinal studies are warranted to confirm these associations and clarify the temporal relationship between systemic inflammation and organ-specific manifestations in pediatric Behçet’s disease.

## Supplementary Material

supplementary table 1.docx

## Data Availability

The datasets generated and/or analysed during the current study are not publicly available due to ethical and legal restrictions related to patient confidentiality, but are available from the corresponding author on reasonable request.
