# Supplementary material for: Predictive role of inflammatory indexes in systemic manifestations of pediatric Behçet’s disease
Source: Ann Med. 2025 Dec 28;58(1):2604893. doi: 10.1080/07853890.2025.2604893 (PMC12777842; doi:10.1080/07853890.2025.2604893)
Supplement: supplementary table 1.docx [file IANN_A_2604893_SM8103.docx]

**Supplementary Table 1. Minor Elective Surgical Procedures in the Control Group (n=41)**

| **Procedure Type** | **Number of Patients (n)** | **Percentage (%)** |
| --- | --- | --- |
| Tonsillectomy | 10 | 24.4 |
| Adenoidectomy | 8 | 19.5 |
| Circumcision | 6 | 14.6 |
| Hernia repair | 7 | 17.1 |
| Skin lesion excision | 5 | 12.2 |
| Other minor procedures* | 5 | 12.2 |
| **Total** | **41** | **100** |

***** *Other minor procedures included simple soft-tissue excisions, minor dermatologic interventions, and uncomplicated outpatient surgical procedures*.
